# Metabolomic Study of Heterotrophically Grown *Chlorella* sp. Isolated from Wastewater in Northern Sweden

**DOI:** 10.3390/molecules26092410

**Published:** 2021-04-21

**Authors:** Jean Claude Nzayisenga, Anita Sellstedt

**Affiliations:** Department of Plant Physiology, UPSC, Umeå University, 901 87 Umeå, Sweden; jean.claude.nzayisenga@umu.se

**Keywords:** autotrophy, biodiesel, *Chlorella* sp., fatty acids, heterotrophy, metabolomics

## Abstract

There are numerous strains of *Chlorella* with a corresponding variety of metabolic pathways. A strain we previously isolated from wastewater in northern Sweden can grow heterotrophically as well as autotrophically in light and has higher lipid contents under heterotrophic growth conditions. The aims of the present study were to characterize metabolic changes associated with the higher lipid contents in order to enhance our understanding of lipid production in microalgae and potentially identify new compounds with utility in sustainable development. *Inter alia*, the amino acids glutamine and lysine were 7-fold more abundant under heterotrophic conditions, the key metabolic intermediate alpha-ketoglutarate was more abundant under heterotrophic conditions with glucose, and maltose was more abundant under heterotrophic conditions with glycerol than under autotrophic conditions. The metabolite 3-hydroxy-butyric acid, the direct precursor of the biodegradable plastic PHB (poly-3-hydroxy-butyric acid), was also more abundant under heterotrophic conditions. Our metabolomic analysis has provided new insights into the alga’s lipid production pathways and identified metabolites with potential use in sustainable development, such as the production of renewable, biodegradable plastics, cosmetics, and nutraceuticals, with reduced pollution and improvements in both ecological and human health.

## 1. Introduction

Microalgae can play important roles in sustainable development and amelioration of climate change [1,2], both as potential sources of renewable fuel (biodiesel) and substances such as the biodegradable plastic polyhydroxy butyric acid (PHB, from poly-3-hydroxy-butyric acid). There is a direct comparison that can be drawn between micro particles from plastic and diesel particles. Diesel engines exhaust small particles that are less than 2.5 μm [3]. These particles are capable of crossing cell membranes and triggering oxidative stress and inflammation that have been linked to increased risk of cardiovascular and respiratory diseases and lung cancer [4]. Taking this into account, it is urgent to decrease the use of plastics from oil-based materials by increasing the use of products from microalgae.

Microalgae frequently live in varying environments of light, salinity, and temperature. Thus, the production of algal-derived, biologically active compounds may be determined by the selection of appropriate cultivation conditions, making these algae natural bioreactors. Cultivation methods were reviewed [5], and it was found that the carbon assimilation and energy dissipation pathways strongly differ between autotrophically, mixotrophically, and heterotrophically grown algae. Thus, for example, the heterotrophic growth on glucose of *C. vulgaris* is reportedly superior to both autotrophic and mixotrophic cultivation for such purposes [6], with particularly high productivity of biodiesel and other biorefinery products [5]. We previously identified a strain of *Chlorella* that had a high lipid content (39.5%), good biodiesel quality, and low carbohydrate contents (8.2%) when grown under heterotrophic conditions with glycerol as the carbon source. However, the alga’s carbohydrate contents were higher under autotrophic and mixotrophic conditions; thus, an appropriate modulation of growth conditions could be used to enhance the production of bioethanol from its biomass [7]. A strain of *C. zofingiensis* reportedly has higher contents of total lipids, neutral lipids, triacylglycerols (TAGs, key precursors of biodiesel production), and oleic acid under heterotrophic conditions [8]. In stark contrast, ref. [9] found that the lipid content of the yellow-green alga *Tribonema minus* was much higher under autotrophic conditions than under heterotrophic conditions. Of course, diverse variables influence lipid production, and attempts to improve the financial viability of biofuel production from some microalgae have been shown. *Inter alia*, nitrogen starvation can substantially increase their TAG generation; thus, this is the most common approach for enhancing their lipid production [10]. The concentration of one precursor of biodiesel production, namely triacylglycerol, was shown to significantly increase in certain microalgae species upon nitrogen starvation [10]. All these studies described different aspects of algae under different growth conditions. However, the understanding of these variations is currently hindered by a paucity of detailed metabolomic information on heterotrophically grown algae [11], relative to the vast amounts of metabolomic information available on autotrophically and mixotrophically grown algae [12,13]. This prompted us to compare metabolomic profiles of the *Chlorella* strain we previously isolated under heterotrophic and autotroph growth conditions. We also compared cultures of the alga grown with both glucose and glycerol as the sole carbon source, as the former is widely used for this purpose, but the latter is cheaper [14,15]. Glycerol is absorbed by algae that can use it as a carbon source via simple diffusion, then transformed by reaction with ATP into glyceraldehyde-3-phosphate, an intermediate in glycolysis, and subsequently triose phosphate. The mechanisms and enzymes involved are well understood [5], but the mechanisms involved in the higher lipid production under heterotrophic conditions with glycerol as the carbon source remain unclear. Thus, the main aims of the study presented here were to characterize the metabolic changes associated with the alga’s higher lipid contents under heterotrophic growth conditions. Such information is important for modulating lipid production in microalgae and hence the production of diverse substances with potential utility in sustainable development, with benefits for both ecological and human health.

## 2. Materials and Methods

### 2.1. Growth Assessment

To examine the effects of the growth conditions on the alga’s biomass, sets of eight cultures of the locally isolated microalga strain *Chlorella* sp. [7] were grown in autotrophic, mixotrophic, and heterotrophic conditions for 8 days. In all cases, the basal growth medium was municipal wastewater collected and immediately stored at 4 °C for at most 10 days. Thereafter, the wastewater was filtered, autoclaved, and otherwise treated as previously described [7]. Algal inoculum was grown in 100 mL portions of BG11 in 500 mL flasks under autotrophic conditions (16 h light/8 h dark cycles, with 120 μmol/m^2^ s^−1^ illumination during the light phase and constant bubbling with 5% CO_2_, at 25 °C). To start each growth experiment, 200 mL of wastewater in a 1 l flask was inoculated with inoculum to an optical density (OD_630_) of 0.06 (equivalent to 0.005 g algal biomass). In autotrophic and mixotrophic conditions, cultures were bubbled with 5% CO_2_, and both mixotrophic and heterotrophic cultures were supplemented with either glycerol or glucose (VWR International, Pennsylvania, PA, USA) to a final concentration of 37.5 mM.

### 2.2. Harvesting

The biomass was collected after 8 days of growth, and after washing twice with distilled water, the cells were recovered by centrifugation (3700× *g*, 6 min), immediately placed in liquid N_2_, then freeze-dried for 3 days and weighed.

### 2.3. Sample Preparation for Metabolomic Analysis

Samples were prepared as previously described [16]. Briefly, 1 mL of extraction buffer (20:20:60 *v*/*v* chloroform:water:methanol) including internal standards was added to 9–12 mg of each sample. The samples were shaken with a tungsten bead in a mixer mill at 30 Hz for 3 min, the bead was removed, and the sample was centrifuged at +4 °C, 14,000 rpm, for 10 min. Next, 200 µL of supernatant was transferred to a micro vial, and solvents were evaporated.

### 2.4. Derivatization

Derivatization was performed as earlier described [16]. Briefly, 30 µL of methoxyamine (15 µg/µL in pyridine) were added to the dry sample, which was shaken vigorously for 10 min before left to react at room temperature for 16 h. Following addition of 30 µL of *N*-methyl-*N*-(trimethylsilyl)trifluoroacetamide (MSTFA), the samples were shaken and incubated for a further hour at room temperature. A 30 µL portion of methyl stearate solution (15 ng/µL in heptane) was subsequently added, and the samples were subjected to gas chromatography-mass spectrometry (GC-MS, Agilent 7890, Santa Clara, CA, USA) analysis, as previously described [16,17]. Briefly, 1 µL of the derivatized sample was injected in either splitless or split (1:20) mode by an CTC Combi Pal Xt Duo (CTC Analytics AG, Zwingen, Switzerland) autosampler into an Agilent 7890A gas chromatograph equipped with a 30 m × 0.25 mm i.d. fused-silica capillary column with a chemically bonded 0.25 μm DB 5-MS UI stationary phase (J&W Scientific, Folsom, CA, USA). The voltage was turned on after a 290 s solvent delay.

### 2.5. Data Evaluation

Raw MS files from the metabolic analyses were exported from ChromaTOF software (LECO, St Joseph, MI, USA) in NetCDF format to Matlab™ (Mathworks, Natick, MA, USA). Following pre-treatment procedures, such as baseline correction and chromatogram alignment in Matlab, the data were further processed by peak integration and multivariate curve resolution using custom scripts. Next, analytes were identified by comparison of their retention time indices and mass spectra with those of entries in both in-house and public databases.

Finally, differences between cultures grown under different conditions were explored by principal coordinate analysis (PCA) using SIMCA (Umetrics, Umeå, Sweden). The differences between algae grown heterotrophically with glycerol, heterotrophically with glycerol, autotrophically, and mixotrophically were analysed by *t*-tests.

## 3. Results and Discussion

Algal heterotrophic metabolism can occur in the light as well as the dark [18]. However, the metabolic pathways involved differ and require elucidation in order to optimize algal exploitation for production of biodiesel and/or other substances. For example, reduced power and energy (ATP) are obtained via glycolysis, the pentose phosphate and tricarboxylic acid pathways linked to mitochondrial electron transport pathways, in dark, aerobic conditions, but via photosynthesis in autotrophic conditions [18]. This study provides more detailed information on the metabolic profiles, and hence key pathways, of the local *Chlorella* strain under four conditions: autotrophic, mixotrophic, and heterotrophic with glycerol or glucose as the sole carbon source.

### 3.1. Growth of Chlorella Vulgaris

After 8 days of growth, there was no significant difference in biomass between cultures grown autotrophically, mixotrophically with glycerol, and heterotrophically with glucose (Table 1). However, cells grown mixotrophically with glucose and heterotrophically with glycerol had accumulated less biomass than autotrophic cells (Table 1). These results are consistent with previous findings regarding the growth of *Chlorella* sp. and the amounts of phosphorous and nitrogen taken up by heterotrophic grown cultures [7]. It was shown that the amount of phosphorous and nitrogen taken up by heterotrophically grown cultures with glycerol were 5 and 19 mg·L^−1^, corresponding to 83 and 80% uptake, respectively [7].

### 3.2. Metabolomic Analysis of Chlorella Alga Under Auto-, Mixo-, and Heterotrophic Conditions

**Figure 1:**
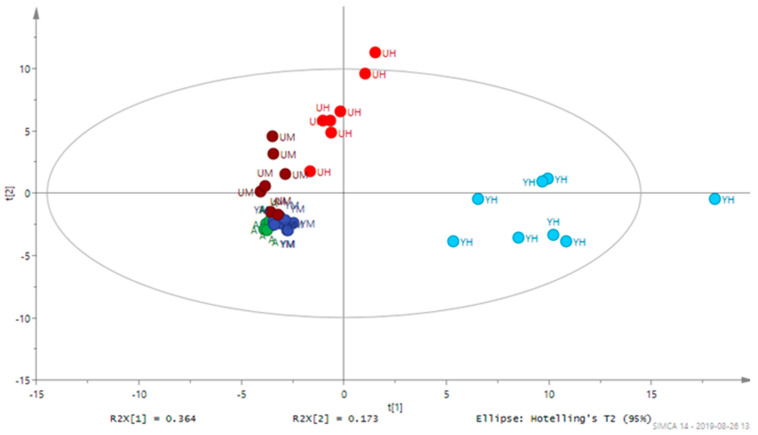
PCA score plot showing metabolomic separation of sets of eight replicates of *Chlorella* cultures after 8 days of cultivation in the following growth conditions: autotrophic (**A**), mixotrophic with glycerol (**YM**), heterotrophic with glycerol (**YH**), mixotrophic with glucose (**UM**), heterotrophic with glucose (**UH**). Eight replicates for each growth condition are shown.

The GC-MS analysis identified 74 distinct metabolites that were present in all samples (Table 2). Key metabolites and metabolite classes separating the samples are shown in the constructed traditional principal coordinate analysis PCA plot presented in Figure 1, which clearly reveals correlations between the abundance of metabolites and growth conditions. It shows that the growth conditions substantially influenced the levels of certain metabolites, *inter alia* there were major metabolomic differences between cells grown heterotrophically with glucose and glycerol; thus, it was clearly shown that the samples could be separated based on the growth conditions. Moreover, there were major differences between cells grown heterotrophically in glucose and glycerol (Figure 1).

**Figure 2:**
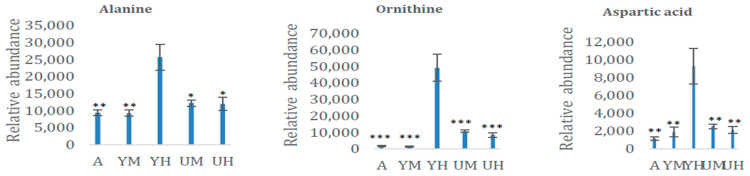
Relative contents (means ± standard deviations, *n* = 8) of three amino acids in the *Chlorella* cultures after 8 days of cultivation in the following growth conditions: autotrophic (AUTO), mixotrophic with glycerol (GLY MIXO), heterotrophic with glycerol (GLY HETE), mixotrophic with glucose (GLU MIXO), heterotrophic with glucose (GLU HETE). *, **, and *** indicate significant differences in concentration relative to cultures grown in heterotrophic conditions with glycerol according to Student’s *t*-test at *p* < 0.05, 0.01, and 0.001, respectively.

**Figure 3:**
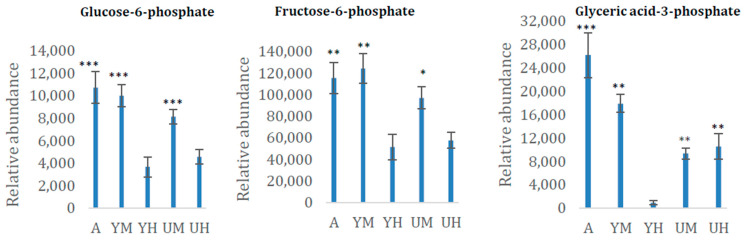
Relative content of three key carbon metabolism intermediates (means ± standard deviations, *n* = 8) in the *Chlorella* cultures after 8 days of cultivation in the following conditions: autotrophic (AUTO), mixotrophic with glycerol (GLY MIXO), heterotrophic with glycerol (GLY HETE), mixotrophic with glucose (GLU MIXO), heterotrophic with glucose (GLU HETE). *, **, and *** indicate significant differences in concentration relative to cultures grown in heterotrophic conditions with glycerol according to Student’s *t*-test at *p* < 0.05, 0.01, and 0.001, respectively.

**Figure 4:**
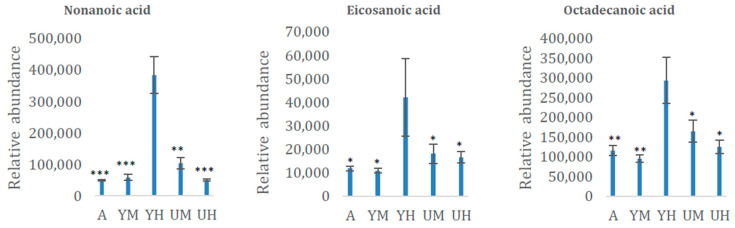
Relative contents (means ± standard deviation, *n* = 8) of three fatty acids in the *Chlorella* cultures after 8 days of cultivation in the following growth conditions: autotrophic (AUTO), mixotrophic with glycerol (GLY MIXO), heterotrophic with glycerol (GLY HETE), mixotrophic with glucose (GLU MIXO), heterotrophic with glucose (GLU HETE). The data are presented as means ± standard deviations, *n* = 8. *, **, *** indicate significant differences in metabolite concentration relative to cultures grown in heterotrophic conditions with glycerol according to Student’s *t*-test, *p* < 0.05, 0.01, and 0.001, respectively.

**Figure 5:**
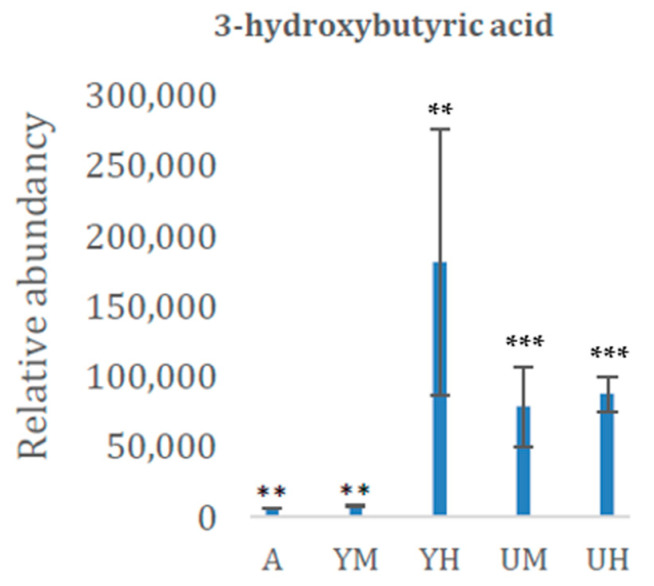
Relative content of 3-hydroxy butyric acid (means ± standard deviations, *n* = 8) in the *Chlorella* cultures after 8 days of cultivation in the following conditions: autotrophic (AUTO), mixotrophic with glycerol (GLY MIXO), heterotrophic with glycerol (GLY HETE), mixotrophic with glucose (GLU MIXO), heterotrophic with glucose (GLU HETE). ** and *** indicate significant differences in concentration relative to cultures grown in heterotrophic conditions with glycerol according to Student’s *t*-test at *p* < 0.01 and 0.001, respectively.

The relative abundances of selected metabolites presented in Figure 2, Figure 3, Figure 4 and Figure 5 clearly show cultivation-condition-related metabolomic variations. The levels of amino acids (notably alanine, ornithine, and aspartic acid) were several times higher following heterotrophic growth with glycerol than under other growth conditions (Figure 2). This has strong physiological (as well as metabolomic) implications because in addition to being building blocks of proteins, amino acids are precursors of diverse N-containing molecules such as nucleic acids, polyamines, quaternary ammonium compounds, and some hormones.

Under environmental stress, de novo protein synthesis is generally inhibited, and protein turnover and proteolytic activity are increased, resulting in increases in the total free amino acid content [9,19]. In addition, ref. [19] also found that the N/C assimilation and distribution pathways related to the glutamate-glutamine system, amino acid (GABA) catabolism and synthesis, and the TCA cycle and glycolysis contribute to the shunting of excess carbon into lipid biosynthesis. 

To extend our understanding of the links between growth conditions and metabolite contents in *Chlorella* sp., we focused particularly on three metabolites that play important roles in carbon metabolism: glucose-6-phosphate, fructose-6-phosphate, and glyceric acid-3-phosphate. All three compounds were much less abundant in cells grown heterotrophically with glycerol than in cells grown autotrophically (Table 2 and Figure 3). In addition, fructose-6-phosphate was the most abundant carbohydrate and much more abundant under autotrophic conditions than under the other conditions (Table 2 and Figure 3). As the formation of fructose-6-phosphate is the rate-limiting step in gluconeogenesis, the low levels of glucose-6-phosphate, fructose-6- phosphate, and glyceric acid-3-phosphate observed under heterotrophy with glycerol as the sole carbon source may be associated with low gluconeogenesis rates. This would explain the alga’s high carbohydrate contents among the microalgae under autotrophic conditions (Figure 3). Fructose-1,6-bisphosphatase, which catalyzes the conversion of fructose-1,6-bisphosphate into fructose-6-phosphate during gluconeogenesis, is also reportedly up-regulated in microalgae with low lipid content and high carbohydrate content [9,20]. This corroborates the indication that the higher carbohydrate contents observed under all the conditions except heterotrophy with glycerol are associated with higher contents of sugars involved in glucose synthesis.

Interestingly, several fatty acids (e.g., eicosanoid, hetadecanoic, nonanoic, arachidic, and octadecanoic acids) and the total fatty acid content were more abundant under heterotrophic conditions with glycerol than under autotrophic conditions (Table 2; Figure 4). These results support our previous finding of a correlation between heterotrophic conditions with glycerol as the carbon source and high lipid production [7]. We have also recently observed an association between high linolenic acid (18:3) content and low oleic acid (18:1) content under heterotrophic conditions with glycerol, which improves biodiesel quality [7]. Increased levels of fatty acids such as nonanoic acid (17:0) and arachidic acid (20:0) (Table 2) support our previous results that heterotrophic conditions with glycerol as the carbon source are associated with increased lipid production [7].

A metabolite of particular interest that we detected is 3-hydroxy-butyric acid (Table 2; Figure 5), which is generally obtained from renewable carbohydrates by fermentation [1,2]. Together with component polyhydroxy-alkanoates (PHAs) and starch-, cellulose-, and protein-based substances, this is a major candidate for commercial bioplastic production [21,22]. Moreover, we have previously shown that the local *Chlorella* sp. can produce more lipids (including 18:1 fatty acid), which is important for biodiesel quality [7]. Similarly, [13] found that illumination significantly affected the fatty acid saturation level in the alga *Euglena gracilis*, and that while it could accumulate considerable amounts of short-chain fatty acids in the dark, its desaturase activity (and hence unsaturated fatty acid content) was higher in the light.

### 3.3. Bioplastics

Commonly used plastics originating from oil-based materials are estimated to reach 622 MT by year 2034 [23]. Microplastics are either produced by design or are formed as a result of degradation of macroplastics. An increasing amount of evidence suggests a widespread exposure to microplastics from foods, drinking water, and air [3]. Replacing microplastics with bioplastics produced from algae would be beneficial for human health. Despite a rapid increase in the bioplastic production, it was still only 2.11 MT in 2018 [23]. The component 3-hydroxy-butyric acid is, together with polyhydroxy-alkanoates (PHAs) and starch-, cellulose-, and protein-based substances, a major candidate for commercial bioplastic production [21,22]. It was found in higher amounts under heterotrophic conditions with glycerol in this study (Figure 5).

## 4. Conclusions

In this study, we have identified key metabolites connected to the high lipid contents of a *Chlorella* strain (isolated in northern Sweden) when grown under heterotrophic conditions with glycerol as the sole carbon source. Under all the other growth conditions, we observed higher contents of glucose-6-phosphate, fructose-6-phosphate, and glyceric acid-3-phosphate, which may be associated with higher gluconeogenesis rates. Growth under heterotrophic conditions with glycerol resulted in the highest levels of certain amino acids, for reasons that are unclear and warrant further attention. Moreover, levels of 3-hydroxybutyric acid, which can be potentially used for bioplastic production, were highest under heterotrophic conditions. Thus, these findings may enhance our understanding of lipid production in microalgae and facilitate their biorefinery-based exploitation, with substantial ecological and human health benefits.

## Figures and Tables

**Table 1:** Accumulated biomass of the *Chlorella* strain after 8 days of growth under conditions of cultivation: autotrophic (A), mixotrophic with glycerol (Y M), heterotrophic with glycerol (Y H), mixotrophic with glucose (U M), heterotrophic with glucose (U H). Means ± standard deviations, *n* = 8.

Growth Condition	
A	1.25 ± 0.09
Y M	1.31 ± 0.10
Y H	0.52 ± 0.03
U H	1.29 ± 180
U M	0.70 ± 0.05

**Table 2:** Metabolites detected in *Chlorella* strain and their retention indexes (RI), compound classes (CC; FA = fatty acids; CA = carboxylic acids; AA = amino acids; EC = energy compound; V = vitamins; P = phenols; ACA = amino carboxylic acid; Carb= carbohydrates; AlA = alkylamines; Carb= carbohydrate; AM = amides; SP = sugar phosphates; L = lipids) and P values. The P values of differences in levels of the metabolites between algae grown in autotrophic and heterotrophic conditions with glycerol are according to *t*-tests. Mean ± standard errors, *n* = 8.

Compounds	RI	C.C.	*p* Value
1-myristoylglycerol	2382	FA	<0.0001
2-methylmalic acid	1460	CA	<0.0001
2-oxoisocaproic acid	1207	FA	0.2423
3-hydroxybutyric acid	1156	CA	0.0377
3-hydroxyisobutyric acid	1156	CA	0.0065
4-aminobutyric acid	1522	CA	0.0860
4-hydroxyphenylacetic acid	1636	CA	0.0064
5-hydroxypipecolic acid	1588	CA	0.0139
Adenosine-5-monophosphate	3050	EC	0.0108
Alanine	1100	AA	0.0010
Alpha-ketoglutaric acid	1565	CA	0.0638
Alpha-tocopherol	3145	V	0.5274
Aminomalonic acid	1458	ACA	0.0425
Arabinose/ribose/xylose	1672	CA	<0.0001
Arabitol	1697	SA	0.0002
Asparagine	1658	AA	0.0696
Aspartic acid	1507	AA	0.0011
Benzoic acid	1252	P	0.0420
Beta-alanine	1419	AA	0.0543
Cadaverine	1831	AlA	<0.0001
Citric acid	1800	CA	<0.0001
Dehydroascorbic acid dimer	1837	V	0.0002
Disaccharide	2515	Carb	0.0303
Eicosanoic acid	2435	FA	0.0388
Ethanolamine	1260	AA	0.0003
Fructose-6-phosphate	2280	SP	0.0041
Fumaric acid	1342	CA	0.0112
Glucose	1876	S	0.0393
Glucose-6-phosphate	2316/2327	SP	0.0008
Glutamic acid	1607	AA	0.0485
Glutamine	1763	AA	0.0438
Glyceric acid	1318	CA	<0.0001
Glyceric acid-3-phosphate	1787	CA	<0.0001
Glycerol	1267	SA	<0.0001
Glycerol-2-phosphate	1704	SP	0.0001
Glycerol-3-phosphate	1743	SP	<0.0001
Glycine	1120	AA	0.8101
Glycolic acid	1074	CA	0.0004
Heptadecanoic acid	2139	L	0.0052
Hexadecanoic acid	2042	L	0.0616
Inositol, myo-	2074	V	0.0151
Inositol, scyllo-	2008	V	<0.0001
Inositol-phosphate	2396	V	0.3207
Isoerythritol	1479	S	0.0336
Lactic acid	1057	CA	0.1729
Lysine	1848	AA	0.0232
Lyxose	1633	S	0.0059
Malic acid	1474	CA	0.6389
Maltose/turanose	2727	S	<0.0001
Methylsuccinate	1320	CA	<0.0001
Myristic acid 50	1844	FA	0.0027
Nicotinamide	1844	V	0.0002
Nonanoic acid	1357	FA	<0.0001
Octadecatrienoic acid, 6,9,12-(z,z,z)-	2191	FA	0.061
Octadecatrienoic acid, 9,12,15-(z,z,z)-	2218	FA	0.0370
*O*-phosphoetanolamine	1771	AM	<0.0001
Ornithine	1453	AA	<0.0001
Pantothenic acid	1980	V	0.0043
Phenylalanine	1680	A	0.0036
Putrescine	1732	AM	0.0768
Pyroglutamic acid	1518	AA	0.0315
Pyruvic acid	1049	CA	<0.0001
Raffinose	3373	S	0.0062
Salicin	2488	S	<0.0001
Succinic acid	1308	S	<0.0001
Sucrose	2616	S	0.2146
Threonine/allo-threonine	1372	AA	0.027205
Trehalose/maltose	2721	S	<0.0001
Tyrosine	1933	AA	0.6952
Valine	1208	AA	0.0455
Xylitol	1684	S	0.0050
Xylulose	1654	S	<0.0001

## Data Availability

Not applicable.

## Sample Availability

Samples of the compounds are not available from the authors.
